# 642. Facility Reported vs. CLSI MIC Breakpoint Comparison of Carbapenem Non-susceptible (Carb-NS) Enterobacteriaceae (ENT) from 2016-2019: A Multicenter Evaluation

**DOI:** 10.1093/ofid/ofab466.839

**Published:** 2021-12-04

**Authors:** Vikas Gupta, Kalvin Yu, Jason M Pogue, Janet Weeks, Cornelius J Clancy

**Affiliations:** 1 Becton, Dickinson and Company, Franklin Lakes, NJ; 2 College of Pharmacy, University of Michigan, Ann Arbor, MI; 3 University of Pittsburgh, Pittsburgh, PA

## Abstract

**Background:**

Carbapenem (Carb) minimum inhibitory concentration (MIC) breakpoints were lowered by CLSI in 2010 and recognized by FDA in 2012. Adoption of revised breakpoints is often slow, which may lead to under-reporting of Carb non-susceptibility (NS) by facilities. We compare facility-reported rates of Carb-NS ENT to the CLSI MIC breakpoints for a large nationwide collection of isolates in the United States (US) from 2016-2019.

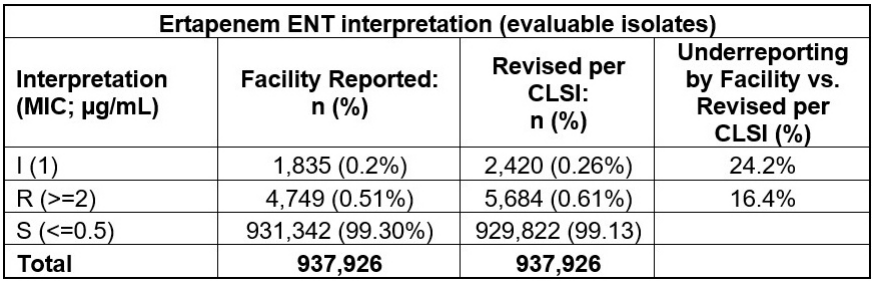

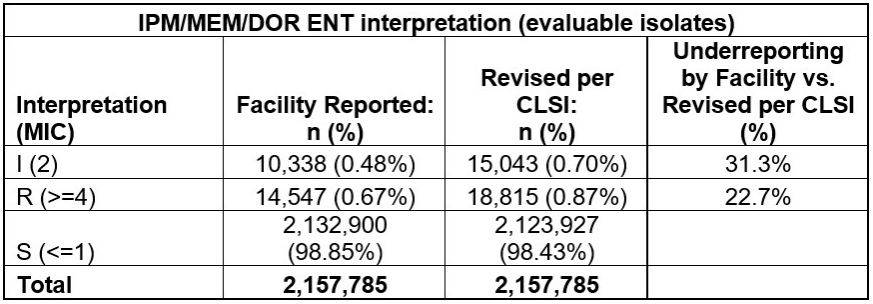

**Methods:**

All adults with a positive non-contaminant ENT culture (first isolate of a species per 30-day period from blood, respiratory, urine, skin/wound, intra-abdominal, or other) in ambulatory/inpatient settings from up to 300 US hospitals from 2016-2019 were evaluated (BD Insights Research Database). Facility-reported Carb-NS was defined as: susceptible (S), intermediate (I) or R to ertapenem (ETP), imipenem (IPM), meropenem (MEM) and/or doripenem (DOR) per commercial panels. Where available, MICs were interpreted using CLSI 2010 MIC breakpoints (µg/ml): ≤ 0.5 (S), 1 (I), ≥ 2 (R) for ETP and ≤1 (S), 2 (I), and ≥ 4 (R) for IPM/MEM/DOR. For evaluable ENT isolates we compared susceptibility results as reported by the facility to CLSI MIC breakpoints.

**Results:**

Overall, 77.4% (937,926/1,211,845) and 90.6% (2,157,785/2,381,824) non-duplicate ENT isolates with facility-reported susceptibility results also had interpretable MIC results for ETP and IPM/MEM/DOR, respectively (Tables). ETP S rates were 99.3% and 99.1% as reported by facilities and using CLSI criteria, respectively. S rates of other Carbs were 98.9% and 98.4% by facility reporting and CLSI criteria, respectively. Systematic application of CLSI breakpoints under-reported EPT-I and –R isolates by 24.2% and 16.4%, respectively, and identification of IPM/MEM/DOR-I and –R isolates by 31.3% and 22.7%, respectively.

**Conclusion:**

Systematic application of CLSI breakpoints in 2016-19 would have had minimal impact on ENT S rates in the US. However, facility reporting failed to identify 18.8% of ETP I or R and 26.5% of IPM/MEM/DOR I or R isolates. The clinical implications of this observation are unknown. Facilities should know their local epidemiology, decide if under-reporting might be an issue, and assess if there is any impact on their patients.

**Disclosures:**

**Vikas Gupta, PharmD, BCPS**, **Becton, Dickinson and Company** (Employee, Shareholder) **Kalvin Yu, MD**, **BD** (Employee) **Jason M Pogue, PharmD, BCPS, BCIDP**, **Merck** (Consultant)**QPex** (Consultant)**Shionogi** (Consultant)**Utility Therapeutics** (Consultant)**VenatoRX** (Consultant) **Janet Weeks, PhD**, **Becton, Dickinson and Company** (Employee) **Cornelius J. Clancy, MD**, **Merck** (Grant/Research Support)

